# Exploring lipodystrophy gene expression in adipocytes: unveiling insights into the pathogenesis of insulin resistance, type 2 diabetes, and clustering diseases (metabolic syndrome) in Asian Indians

**DOI:** 10.3389/fendo.2024.1468824

**Published:** 2024-10-09

**Authors:** Aditya Saxena, Pradeep Tiwari, Shalu Gupta, Rajendra Mandia, Ramesh C. Banshiwal, Ravinder Kumar Lamoria, Ranjit Mohan Anjana, Venkatesan Radha, Viswanathan Mohan, Sandeep Kumar Mathur

**Affiliations:** ^1^ Department of Computer Engineering & Applications, GLA University, Mathura, India; ^2^ Department of Biotechnology and Bioinformatics, Birla Institute of Scientific Research, Jaipur, India; ^3^ Department of General Surgery, Sawai Man Singh (SMS) Medical College and Attached Hospital, Jaipur, India; ^4^ Department of Orthopedics, Sawai Man Singh (SMS) Medical College and Attached Hospital, Jaipur, India; ^5^ Department of Diabetology, Madras Diabetes Research Foundation, Chennai, Tamil Nadu, India; ^6^ Department of Endocrinology, Sawai Man Singh (SMS) Medical College, Jaipur, India

**Keywords:** lipodystrophy, type 2 diabetes, Asian Indians, central obesity, insulin resistance, metabolic syndrome MetS, qPCR, adipose tissue

## Abstract

**Background:**

Studying the molecular mechanisms of lipodystrophy can provide valuable insights into the pathophysiology of insulin resistance (IR), type 2 diabetes (T2D), and other clustering diseases [metabolic syndrome (MetS)] and its underlying adipocentric disease (MetS disease).

**Methods:**

A high-confidence lipodystrophy gene panel comprising 50 genes was created, and their expressions were measured in the visceral and subcutaneous (both peripheral and abdominal) adipose depots of MetS and non-MetS individuals at a tertiary care medical facility.

**Results:**

Most lipodystrophy genes showed significant downregulation in MetS individuals compared to non-MetS individuals in both subcutaneous and visceral depots. In the abdominal compartment, all the genes showed relatively higher expression in visceral depot as compared to their subcutaneous counterpart, and this difference narrowed with increasing severity of MetS. Their expression level shows an inverse correlation with T2D, MetS, and HOMA-IR and with other T2D-related intermediate traits. Results also demonstrated that individualization of MetS patients could be done based on adipose tissue expression of just 12 genes.

**Conclusion:**

Adipose tissue expression of lipodystrophy genes shows an association with MetS and its intermediate phenotypic traits. Mutations of these genes are known to cause congenital lipodystrophy syndromes, whereas their altered expression in adipose tissue contributes to the pathogenesis of IR, T2D, and MetS.

## Introduction

Metabolic syndrome (MetS) has long been debated as a predictor of atherosclerotic cardiovascular (CV) risk, but it has not been universally accepted as a clinical entity. It potentially offers no additional CV risk beyond its individual components, and its unifying pathophysiological mechanisms remain largely unexplored (1–3). Nevertheless, MetS is recognized as a complex condition characterized by metabolic dysregulation, including insulin resistance (IR), hyperinsulinemia, and increased susceptibility to type 2 diabetes (T2D), cardiovascular disease (CVD), hypertension, Alzheimer’s disease, certain cancers, and other disorders (4, 5). Individuals with MetS may also exhibit low-grade inflammation, oxidative and endoplasmic reticulum (ER) stress, mitochondrial dysfunction, and impaired exercise capacity (6–8). It is commonly associated with obesity, particularly central obesity, but it can also involve subtle manifestations of excess adiposity, such as ectopic fat deposits in the liver, muscle, pancreas, etc. (9–11). Therefore, these insights into its pathophysiology point toward an adipocentric metabolic disorder that is clinically expressed as IR and other clustering diseases. In this article, we propose to coin the term “MetS disease or adiposopathy” to describe this adipocentric metabolic disorder underlying a cluster of diseases, the so-called “clinical MetS”.

Phenotypically, MetS bears a resemblance to a rare genetic disease, i.e., congenital lipodystrophy syndrome, although it has less severe clinical manifestations (12–16). They depict a U-shaped relationship between adiposity and consequent morbidity and mortality (“obesity-paradox”) (3–6). Interestingly, many South Asians with T2D and MetS illustrate this obesity paradox because dysmetabolism in them is associated with a relatively low body mass index (BMI) (7–11). Although traditionally IR and T2D in them is attributed to increased visceral fat accumulation, recent observations suggest that it is associated with less peripheral fat and more ectopic liver fat. IR in them shows a trend toward stronger positive and negative correlation with ectopic liver and subcutaneous fat mass respectively in its higher quartiles (17). This fat distribution pattern mimics that of congenital partial lipodystrophy syndromes (18–20).

Congenital lipodystrophies are monogenic genetic diseases in which the underlying genetic mutations, the consequent molecular mechanisms leading to adipose tissue deficiency or severe dysfunction, and the pathophysiology of metabolic dysfunction are well-deciphered (14, 21, 22). In contrast, T2D and MetS are genetically complex traits in which genome-to-phenome pathways remain poorly understood despite numerous genome-wide association studies (GWAS) (23–31). Interestingly, genetic risk scores for IR and associated loci are linked to lower BMI and lesser gluteal–femoral and peripheral fat mass (32–35). Therefore, these findings suggest a role for genetically determined relative adipose deficiency in the pathogenesis of dysmetabolism, which is similar to that of congenital lipodystrophy syndromes. The role of genetically determined adipose dysfunction in the pathogenesis of IR, T2D, and MetS in South Asians is suggested by the findings of adipose tissue transcriptomic studies, in which modules of co-expressed genes of differentially expressed genes converge on the pathways of adipogenesis and inflammation (7, 11, 36–46). Additionally, these modules of co-expressed genes show an association with T2D and its intermediate phenotypic traits (47, 48). This adipocentric molecular pathophysiology is similar to that of various congenital lipodystrophy syndromes.

Given these parallels in clinical phenotype, adipose tissue dysfunction, and the partly understood genetic predisposition and pathways, insights gained from studying lipodystrophy mechanisms could aid in decoding the complex genetic traits of IR, T2D, and MetS disease (18, 19, 36). We previously observed altered expression of lipodystrophy genes in the peripheral subcutaneous adipose tissue of individuals with T2D (49). These findings suggest a potential role for the functional defects of these genes in the pathogenesis of IR, T2D, and possibly MetS. However, the role of altered expression of lipodystrophy genes in different adipose tissue depots in the pathogenesis of IR, T2D, and MetS remains largely unexplored. Therefore, this study investigated whether “clinical MetS” and T2D are associated with altered expression of lipodystrophy genes in various adipose tissue depots. Second, do their expression levels correlate with T2D- and MetS-related intermediate phenotypic traits such as β-cell function and IR? This approach may provide insights into the functional roles of these genes in the pathogenesis of T2D and MetS, potentially offering new predictive and therapeutic strategies.

We therefore conducted the present study to decipher the probable link between a selected set of 50 lipodystrophy genes with T2D and MetS pathophysiology by measuring their expression in abdominal and femoral adipose tissue using qPCR. Additionally, we attempted to classify the clinical severity of “clinical MetS” based on lipodystrophy gene expression using a machine learning technique—decision tree—to personalize patient care. In recent years, there has been a noticeable rise in the application of machine learning and deep learning in biomedical fields (50, 51). However, the use of decision tree models to stratify patients according to disease-specific gene expression remains underutilized and could therefore be considered a promising approach for future patient care.

## Materials and methods

### Study population

The study was conducted at Sawai Man Singh Medical College, Jaipur, India. Based on the anatomical site for the collection of adipose tissue biopsies, subjects were divided into two cohorts: abdominal–visceral fat and subcutaneous fat. A total of 78 individuals, comprising 43 non-MetS controls and 35 MetS patients undergoing abdominal surgery, were recruited, with paired samples taken from each subject—one from the subcutaneous compartment and one from the visceral adipose depot. Additionally, 69 individuals, comprising 50 non-MetS controls and 19 MetS patients undergoing femur bone surgery for traumatic fractures, were recruited, and adipose tissue biopsies were collected. The visceral cohort contained 78 biopsies, while the subcutaneous cohort contained 147 biopsies (78 from the abdominal surgery group and 69 from the femur bone surgery group).

All methods were performed as per the relevant guidelines and regulations. The inclusion criteria for MetS participants were as per NCEP ATP III standards. The exclusion criteria were the presence of infection, malignancy, and drugs acting on body fat/IR or adipocytokine expression such as thiazolidinediones, metformin, and glucocorticoids. Our study examined male and female individuals, and similar findings are reported for both genders. Institutional ethics committee approval of the study was obtained and all the participants provided written consent.

### Clinical and biochemical assessment

The anthropometric measurements, including body weight, height, waist-to-hip (W: H) ratio, and BMI, were obtained using standard methods on all samples. Supine blood pressure was measured using a mercury sphygmomanometer (BPMR-120 Diamond deluxe, Industrial electronic and allied products, Maharashtra, India) after 10 min of rest. Blood samples were obtained at 8:00 a.m. after an overnight fast of at least 8 h. Various biochemical parameters [e.g., serum glucose, lipid profile, triglycerides, low-density lipoprotein cholesterol (LDL-C), high-density lipoprotein cholesterols (HDL-C), and very-low-density lipoprotein cholesterol (VLDL-C)] were measured on a Kopran AU/400 (Olympus Corporation, Shinjuku, Tokyo, Japan) fully automated analyzer. Serum insulin was measured using a chemiluminescent immunometric assay (Immulite 2000 machine, Siemens Healthineers AG, Erlangen, Germany). HbA1c was measured by a turbidimetry method using BioSystems (BioSystems, SA Barcelona, Spain) kits.

HOMA-β was calculated using the following formula:


360×[Insulin in μU/mL]/([Glucose in mg/dL]−63)

HOMA-IR is a measure of insulin resistance, and was calculated using the following formula:


{[Clucose in mg/dL]×[Insulin in μU/mL]}/405

### Radiological investigations

Total body and regional fat content and distribution of participants were estimated by dual-energy x-ray absorptiometry (DEXA) using the Hologic Explorer model (S/N91395 make, Hologic Canada ULC, Mississauga, Ontario, Canada). Abdominal fat content and distribution among viscera, subcutaneous, and ectopic hepatic compartments was estimated by MRI.

MRI scan procedure: The MRI scans were done at the Department of Radiology of the SMS Hospital, using Philips Ingenia Machine of 3 T. The observer and the radiologist who interpreted the scans were unaware of the clinical status of the study subjects. A single scan (10 mm) of the abdomen was done at the level of L4–L5 vertebrae and analyzed for a cross-sectional area of adipose tissue, which was expressed in centimeters squared. Areas were calculated by multiplying the number of pixels of a given tissue type by the pixel number (pixel density). The parameters studied included visceral and subcutaneous fat. Visceral fat was distinguished from subcutaneous abdominal fat by tracing along the fascial plane defining the internal abdominal wall. Liver fat intensity was also measured using liver intensity.

### Adipose tissue cell size measurement

All the biopsies taken in formalin solution were processed for making tissue blocks and slides for determining the area of adipocytes using standard protocols. Imaging of adipocytes was done at 10× magnification using a Motic Panthera Moticam 5 trinocular microscope (BA210LED) (Motic Incorporation Ltd., Hong Kong, China). The area of adipocytes was measured using ImageJ (http://imagej.nih.gov/ij) image analysis tool in µm^2^.

### Selection of lipodystrophy genes for qPCR investigation

A total of 267 genes were implicated in lipodystrophy along with its 26 variants (such as acquired, partial, generalized, familial, genetic, congenital, and HIV-associated) in the DisGeNET database (52). These genes constituted our lipodystrophy gene population for subsequent gene selection for qPCR analysis.

We used a four-metric approach devised by DisGeNET for the quantification of disease–gene association by computing a combined rank for each gene across its values of Disease Specificity Index (DSI), Disease Pleiotropy Index (DPI), pLI (probability of being loss-of-function intolerant), and the number of lipodystrophy disease variants associated with it.

The DSI reflects if a gene is associated to several or fewer diseases and is computed according to:


$$DSI=\frac{log_{2}\left(\frac{N_{d}}{N_{T}}\right)}{log_{2}\left(\frac{1}{N_{T}}\right)}$$

where *N_d_* is the number of diseases associated to the gene/variant and *N_T_* is the total number of diseases in DisGeNET.

DPI considers if the multiple diseases associated to the gene are similar among them (belong to the same MeSH disease class, e.g., CVDs) or are completely different diseases and belong to different disease classes. DPI is computed according to:


$$DPI=\left(\frac{N_{dc}}{N_{TC}}\right)*100$$

where *N_dc_* is the number of the different MeSH disease classes of the diseases associated to the gene/variant and *N_TC_* is the total number of MeSH diseases classes in DisGeNET.

The pLI score is a probability metric that reflects the observed versus expected number of loss-of-function (LoF) variants in a gene. If a gene associated with a particular disease has a high pLI score, it suggests that the gene is less likely to tolerate loss-of-function variants, and mutations in this gene may contribute to the development of the associated disease.

Based on this approach, we selected 49 genes; the average DSI, DPI, and pLI for selected genes were 0.5, 0.7, and 0.3, respectively, which were comparable with our total lipodystrophy gene population. However, the average of number of lipodystrophy disease variants for selected genes was 3.4, which was higher than the gene population (~1.6).

This panel of 49 genes also included 19 genes from the lipodystrophy panel, which was recommended by the Genetic Testing Registry for the screening, diagnosis, mutation confirmation, pre-symptomatic, monitoring, and risk assessment of congenital generalized lipodystrophy type 1 (https://www.ncbi.nlm.nih.gov/gtr/tests/501102/). A 50th gene, *TBC1D4*, was therefore included to cover the entire panel, and the list of selected genes is presented in **Table 1**.

**Table 1:** List of selected lipodystrophy genes and their primer sequences for qPCR validation.

S. no.	Gene symbol	Name	Primer pairs
1	*PIK3R1*	Phosphoinositide-3-kinase regulatory subunit 1	*Forward, 5′-CTCTGGTTGGTGTGGGCT-3′*
			*Reverse, 5′-AGGAAGAGAGTCGCGGCA-3′*
2	*PPARA*	Peroxisome proliferator activated receptor alpha	*Forward, 5′-GAGGACACACACCGAGGACT-3′*
			*Reverse, 5′-GCAGCTGGAGGAACAAACAC-3′*
3	*PTPRC*	Protein tyrosine phosphatase, receptor type C	*Forward, 5′-GCTGAGTTTTGAATGCCCTAA-3′*
			*Reverse, 5′-AGAGATGACAAACAGATTCAGCA-3′*
4	*LEP*	Leptin	*Forward, 5′-GATCGGGCCGCTATAAGAG-3′*
			*Reverse, 5′-GTCCAGAACTAAGCCATCCG-3′*
5	*ZMPSTE24*	Zinc metallopeptidase STE24	*Forward, 5′-GTGGCAAGCTATAAACCATTCGA-3′*
			*Reverse, 5′-TGAAAACAACCAGACAGACTGT-3′*
6	*B4GALT7*	Beta-1,4-galactosyltransferase 7	*Forward, 5′-CAAGCAGAACCTTGGAGGC-3′*
			*Reverse, 5′-CAGACATGGAGACGTGATGG-3′*
7	*GLMN*	Glomulin, FKBP-associated protein	*Forward, 5′-GGGAGAGTAGTCTGCCGGA-3′*
			*Reverse, 5′-CATGTCTTCCGATTCCTGCT-3′*
8	*LMNA*	Lamin A/C	*Forward, 5′-CATGCCGGGAGTTGTAGTTT-3′*
			*Reverse, 5′-TTCATACCCGCTCTGTTTCC-3′*
9	*PIK3CD*	Phosphatidylinositol-4,5-bisphosphate 3-kinase catalytic delta	*Forward, 5′-AAGTGGGAAGTGGAGTGTGC-3′*
			*Reverse, 5′-CTCTTCCCACCTCCCCTGA-3′*
10	*LMF1*	Lipase maturation factor 1	*Forward, 5′-GCCACGCCCAGCTATTTCT-3′*
			*Reverse, 5′-AGCAGTCAAGCTTCGGAAGG-3′*
11	*PCK1*	Phosphoenolpyruvate carboxykinase 1	*Forward, 5′-CACCCCAACTCGAGGTTCTG-3′*
			*Reverse, 5′-TACCCAGCACACCCATGTTC-3′*
12	*PCYT1A*	Phosphate cytidylyltransferase 1, choline, alpha	*Forward, 5′-TTTCCTCTGAGAGCTGCAGC-3′*
			*Reverse, 5′-CGTGAGGGTATGCTCTGTGG-3′*
13	*LPIN1*	Lipin 1	*Forward, 5′-ACCACCACCTAATTCCAGAACA-3′*
			*Reverse, 5′-AACCACAGCTGGACATGGAG-3′*
14	*ATP6V1A*	ATPase H+ transporting V1 subunit A	*Forward, 5′-ATCCGTAAAAATTCAGGCCC-3′*
			*Reverse, 5′-CTCCTTCCACCCCCTCTATC-3′*
15	*CIDEC*	Cell death inducing DFFA like effector c	*Forward, 5′-GCTTCATGGGGGCTGGAAAT-3′*
			*Reverse, 5′-GCCTCACCCACTTCGTATCC-3′*
16	*SREBF1*	Sterol regulatory element binding transcription factor 1	*Forward, 5′-GCCGTCTATCTGGGAGGG-3′*
			*Reverse, 5′-ACAAAGGCCAGGGAGACAC-3′*
17	*ADRA2A*	Adrenoceptor alpha 2A	*Forward, 5′-ATCCTGGCCTTGGGAGAGAT-3′*
			*Reverse, 5′-TCTCAAAGCAGGTCCGTGTC-3′*
18	*AGPAT2*	1-Acylglycerol-3-phosphate O-acyltransferase 2	*Forward, 5′-CACCTAGCCCTTCCCTGC-3′*
			*Reverse, 5′-GGGAAGCCCAGAAGAAAGTT-3′*
19	*BSCL2*	BSCL2, seipin lipid droplet biogenesis associated	*Forward, 5′-CTCCCCAAACTCCCTCATGG-3′*
			*Reverse, 5′-GCACCCATTTCAGGCAGAAG-3′*
20	*IL7R*	Interleukin 7 receptor	*Forward, 5′-GGAAAATGTCATGCTCCTGG-3′*
			*Reverse, 5′-CATAAAATCTGTATGACCTGCCC-3′*
21	*LPIN2*	Lipin 2	*Forward, 5′-AGAATGTCATCGTTCCTGCCT-3′*
			*Reverse, 5′-TCTCCTTCCTCTTTCAAGAAACCA-3′*
22	*SAT1*	Spermidine/spermine N1-acetyltransferase 1	*Forward, 5′-GAGAGGTCCCACCTCACG-3′*
			*Reverse, 5′-AGCTCAGGGGAACGGAAT-3′*
23	*INSR*	Insulin receptor	*Forward, 5′-ATGAACATCACCCGGGGTTC-3′*
			*Reverse, 5′-GGCACGAGACACTGCTTAGA-3′*
24	*PSMB4*	Proteasome subunit beta 4	*Forward, 5′-CCCATGGTCCCCTTCTTCAG-3′*
			*Reverse, 5′-TTGTCTTCCCTTTCCCCCAC-3′*
25	*FGF21*	Fibroblast growth factor 21	*Forward, 5′-GGGAGAGGTCCTCGAACCA-3′*
			*Reverse, 5′-ATAGGAGGAGCATGGTGGGT-3′*
26	*AKT2*	AKT serine/threonine kinase 2	*Forward, 5′-AGGCGCTGTTGTTATGCTCT-3′*
			*Reverse, 5′-GGTCTGATAAGATGCGGTGG-3′*
27	*CAV1*	Caveolin 1	*Forward, 5′-TAGACCTCCCCTCCCCAAAC-3′*
			*Reverse, 5′-GCACCAGACCCCCTAAATGT-3′*
28	*FOS*	Fos proto-oncogene, AP-1 transcription factor subunit	*Forward, 5′-TCTGAGACAGGAACTGCGAA-3′*
			*Reverse, 5′-CTCATCTACTGGAGCGTCCC-3′*
29	*ACTB*	Actin beta	*Forward, 5′-CCAACCGCGAGAAGATGA-3′*
			*Reverse, 5′-CCAGAGGCGTACAGGGATAG-3′*
30	*PPARG*	Peroxisome proliferator activated receptor gamma	*Forward, 5′-GCCTAGGCTGTGTACATGTGT-3′*
			*Reverse, 5′-AGGGATACTTTTCAATACAAATGCAGT-3′*
31	*CCL2*	C-C motif chemokine ligand 2	*Forward, 5′-CTCTGCCCGCTTTCAATAAG-3′*
			*Reverse, 5′-GGTACCACGTCTGCTTGGAT-3′*
32	*KCNJ6*	Potassium voltage-gated channel subfamily J member 6	*Forward, 5′-GGAAGAAACTGCAGGAGGGG-3′*
			*Reverse, 5′-AGTGACAGTTGTGGTGGTGG-3′*
33	*USP8*	Ubiquitin-specific peptidase 8	*Forward, 5′-GCGGTGGAAGAGAGAGGAGT-3′*
			*Reverse, 5′-AGACTCAAGGTTGGGCCTTT-3′*
34	*FBN1*	Fibrillin 1	*Forward, 5′-TGGCCATCTCTTCCTCTTCT-3′*
			*Reverse, 5′-CCCCATGCAACCAACACAAC-3′*
35	*CCL3*	C-C motif chemokine ligand 3	*Forward, 5′-GCATGACAGCATCACTACGC-3′*
			*Reverse, 5′-AGGGACTGTAACTCCCCTGC-3′*
36	*CDH23*	Cadherin-related 23	*Forward, 5′-AGGTGAGGGAGGGAGCTG-3′*
			*Reverse, 5′-GAGGGGAAGGAGAGGGAAG-3′*
37	*LMNB2*	Lamin B2	*Forward, 5′-ACTGCTGTAGTCCAAGGGGA-3′*
			*Reverse, 5′-GTGCCAGGATCAGGGTGAC-3′*
38	*PSMB8*	Proteasome subunit beta 8	*Forward, 5′-CGTGACACTACTCCCAGCTC-3′*
			*Reverse, 5′-GGGTCAAGGGTCTTCCGAAG-3′*
39	*CXCL8*	C-X-C motif chemokine ligand 8	*Forward, 5′-CATCAGTTGCAAATCGTGGA-3′*
			*Reverse, 5′-TGTTTGTTACCAAAGCATCAAGA-3′*
40	*SUMO1*	Small ubiquitin-like modifier 1	*Forward, 5′-CATTTCCCGCCTTGTCTTT-3′*
			*Reverse, 5′-CTAGCGGCAGGCAGACCT-3′*
41	*LMNB1*	Lamin B1	*Forward, 5′-AATGATTGCCATGTAATTTTTATCACA-3′*
			*Reverse, 5′-AGAACCCCTCCATTCCTTTACA-3′*
42	*CAVIN1*	Caveolae associated protein 1	*Forward, 5′-AAGGTCAGCGTCAACGTGAA-3′*
			*Reverse, 5′-TCTCCCCACCCCAACTCC-3′*
43	*RETN*	Resistin	*Forward, 5′-GGGGGCCCAGGGACTTAT-3′*
			*Reverse, 5′-CATGTGGGGGACAGGGATG-3′*
44	*KRAS*	KRAS proto-oncogene, GTPase	*Forward, 5′-GTACGCCCGTCTGAAGAAGA-3′*
			*Reverse, 5′-CCCTAATTCATTCACTCGCC-3′*
45	*POLD1*	DNA polymerase delta 1, catalytic subunit	*Forward, 5′-TGCAGTCGAACAAGCGGG-3′*
			*Reverse, 5′-GTTCCCTACCAGGCCCACT-3′*
46	*METTL9*	Methyltransferase like 9	*Forward, 5′-CCAGTGGGCAATCTGAACTT-3′*
			*Reverse, 5′-GAAGAAACCAGAGCATCCGA-3′*
47	*LIPE*	Lipase E, hormone-sensitive type	*Forward, 5′-AGGCCTAAATTGGGATGCTT-3′*
			*Reverse, 5′-GAGTCTTCGATTCTGGCTGG-3′*
48	*POLR3A*	RNA polymerase III subunit A	*Forward, 5′-TGTTAGTTTCTAGTTACCTGGGAGT-3′*
			*Reverse, 5′-ACCACAGTGAGCTTTGCCAT-3′*
49	*PLIN1*	Perilipin 1	*Forward, 5′-GAAGTGACCCAGTGGAGCTC-3′*
			*Reverse, 5′-ATCTCCCCTCCCTGCTTCTT-3′*
50	*TBC1D4*	TBC1 domain family member 4	*Forward, 5′-GATTCTCCACCAGGGACACC-3′*
			*Reverse, 5′-AGTCGGAATCCTCTTCGGGA-3′*

Lines of evidence for the involvement of 38 genes in lipodystrophy or its variants were gleaned from PubMed searches and are presented in **Supplementary Table S1**.

### Real-time PCR

A total of 2 μg of RNA was isolated from femoral subcutaneous adipose tissue biopsy and abdominal adipose tissue biopsy using the Qiagen RNeasy Mini Kit (Cat No. 74104). The quantification of samples was done using a microfluidic-based capillary electrophoresis system (Bio-Rad Experion, Bio-Rad Laboratories, Inc., Philadelphia, PA, USA). cDNA synthesis was performed using the QuantiNova Reverse Transcription Kit (Cat No. 205411) and real-time PCR was carried out using the QuantiNova SYBR Green PCR Kit (Cat. No 208052). We performed qPCR (quantitative polymerase chain reaction) on selected genes. Oligos (primers) were constructed from online available tools and synthesized as per appropriate melting temperature (*T*_m_), GC%, synthesized by Integrated DNA Technologies, Inc. (Coralville, Iowa, USA). The primers synthesized were double checked to get amplimer product. The oligonucleotide primer sequences used in the qPCR analysis are listed in **Table 1**. Gene fold expression was calculated by the formula Fold change = 2^−Δ;Δ;CT^ where 2^−Δ;Δ;CT^ = [(C_T_ gene of interest − C_T_ internal control) sample A − (C_T_ gene of interest − C_T_ internal control) sample B)] (Schmittgen et al., 2008).

### Creation of decision tree

To classify patients into different MetS levels based on gene expressions, we used a machine learning method, DecisionTreeClassifier, from the sklearn package in a Python environment. MetS status was set as the target variable, and all the genes were used as features. The gene selected for splitting the dataset was based on the reduction of entropy, which measures the randomness or impurity in the data. This was achieved by calculating the information gain, which represents the decrease in entropy after the split. For a binary classification problem, such as distinguishing between MetS and non-MetS, the entropy *E*(*S*) was calculated as:


$$E(S)=-p1\;log_{2}(p1)-p2\;log_{2}(p2)$$

where *p*1 is the proportion of data points in the dataset belonging to the MetS class and *p*2 is the proportion of data points in the dataset belonging to the non-MetS class.

Library train_test_split from the same package was used to divide samples in the training (80%) as well as in the test set. Maximum depth for tree construction was set to 10 and graphviz library was used to create the decision tree figure for visualization.

## Results

**Figure 1:**
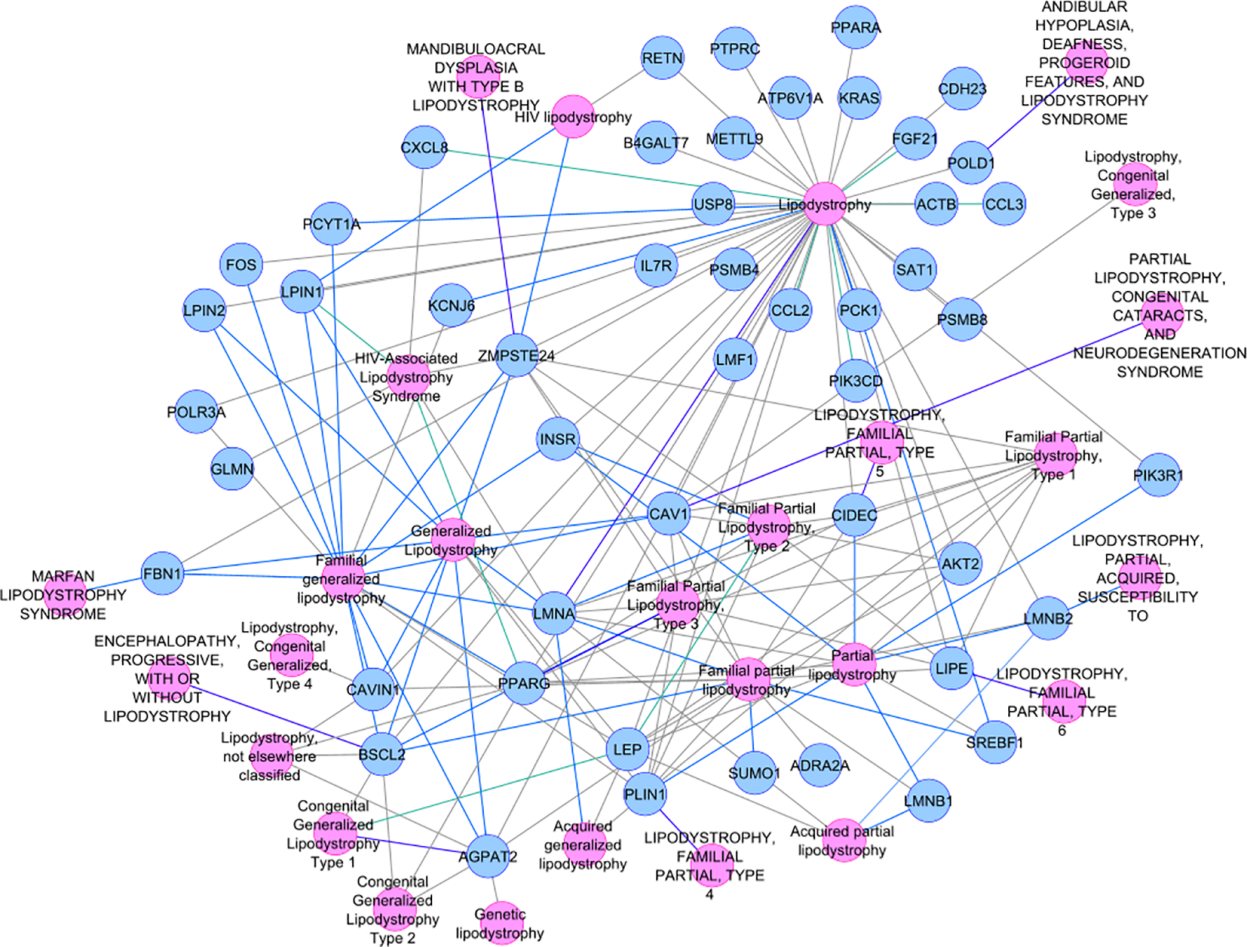
Disease–gene network selected lipodystrophy genes.

Not-MetS and MetS have shown significant differences between their waist circumference and BMI in both thigh and abdominal cohorts. Other glycemic and fat parameters—HOMA-IR, Hb1Ac, and triglycerides—also differed significantly (**Table 2**). Visceral fat mass and adipocyte cell size were found significantly higher in MetS (**Table 3**). For the selected list of 50 lipodystrophy genes, a disease–gene network was constructed using the DisGeNET database in cytoscape environment and they showed an association with lipodystrophy and its variants (**Figure 1**).

**Table 2:** Anthropometric, clinical, and biochemical parameters in subjects with MetS and Non-MetS.

Characteristics	MetS (*n* = 19)	Non-MetS (*n* = 50)	*p*-value	MetS (*n* = 35)	Non-MetS (*n* = 43)	*p*-value
	Thigh cohort (*n* = 69) *t*-test			Abdominal cohort (*n* = 78) *t*-test		
	MetS (*N* = 19)	Non-MetS (*N* = 50)		MetS (*N* = 35)	Non-MetS (*N* = 43)	
Age (years)	51.36 ± 10.78	49.0 ± 14.89	0.46	54.69 ± 9.24	50.45 ± 10.35	0.06
Height (m)	1.60 ± 0.09	1.64 ± 0.10	0.09	1.60 ± 0.08	1.64 ± 0.08	0.06
Weight (kg)	65.05 ± 15.30	62.08 ± 11.91	0.45	61.53 ± 10.42	63.47 ± 10.93	0.44
BMI (kg/m^2^)	25.04 ± 3.88	22.93 ± 3.46	**0.04**	24.26 ± 4.86	23.72 ± 3.80	0.60
Waist (cm)	87.42 ± 8.61	82.72 ± 8.03	**0.04**	93.77 ± 8.57	87.08 ± 8.47	**0.001**
WHR	0.92 ± 0.07	0.90 ± 0.04	0.39	0.98 ± 0.10	0.93 ± 0.07	**0.01**
Total cholesterol (mg/dL)	164.43 ± 56.19	144.2 ± 33.23	0.15	192.74 ± 54.65	166.38 ± 52.74	**0.04**
Triglycerides (mg/dL)	145.89 ± 51.08	110.28 ± 47.56	**0.01**	178.85 ± 65.93	116.08 ± 47.79	**<0.003**
HDL-C (mg/dL)	46.63 ± 16.08	47.18 ± 9.10	0.89	42.52. ± 8.13	46.52 ± 9.88	0.06
LDL-C (mg/dL)	88.63 ± 41.05	73.56 ± 25.19	0.14	109.95 ± 44.77	90.64 ± 34.29	**0.04**
HOMA-IR	5.19 ± 4.26	1.59 ± 1.03	**0.001**	4.71 ± 3.98	1.42 ± 2.64	**<0.001**
Fasting plasma glucose	154.05 ± 83.31	87.76 ± 16.37	**0.002**	170.23 ± 66.07	97.02 ± 35.82	**<0.001**
HbA1c (%)	7.13 ± 2.04	5.06 ± 0.69	**0.003**	7.84 ± 2.16	5.45 ± 1.05	**<0.001**

Significant p-values are shown in bold.

**Table 3:** Abdominal fat mass between MetS and Non-MetS in abdominal cohort.

Abdominal cohort	MetS	Non-MetS	*p-*value (MetS vs. Non-MetS)
Visceral adipose tissue fat (cm^2^)	169.58 ± 54.67	141.43 ± 52.25	**0.04**
Abdominal subcutaneous fat mass (cm^2^)	168.04 ± 69.56	145.25 ± 96.39	0.29
Liver fat (%)	9.02 ± 3.32	7.21 ± 4.08	0.06
Adipocyte size area in µm^2^			
Abdominal visceral adipocyte area	13,385.40 ± 6,093.13	9,759.02 ± 5,782.7232	**0.009**
Abdominal subcutaneous adipocyte area	11,340.60 ± 5,236.25	10,770.98 ± 6,272.22	0.66
Femoral subcutaneous adipocyte area	5,312.63 ± 2,085.01	5,629.54 ± 2,096.67	0.57

Significant p-values are shown in bold.

We also created a T2D interactome by taking 132 predicted T2D effector genes as seed nodes along with their first-degree neighbors from a high-confidence human protein–protein network obtained from ConsensusPathDB and StringDB using the PhenomeScape app (2,222 genes). Interestingly, this network showed ~51% overlap with the interactome of our qPCR (including their first-degree neighbors). This qPCR interactome of 1,191 genes also showed robust enrichment of insulin signaling, adipogenesis, laptin signaling, and AGE/RAGE signaling among others in WikiPathways.

### Identified differentially expressed genes and their co-expression modules

**Figure 2:**
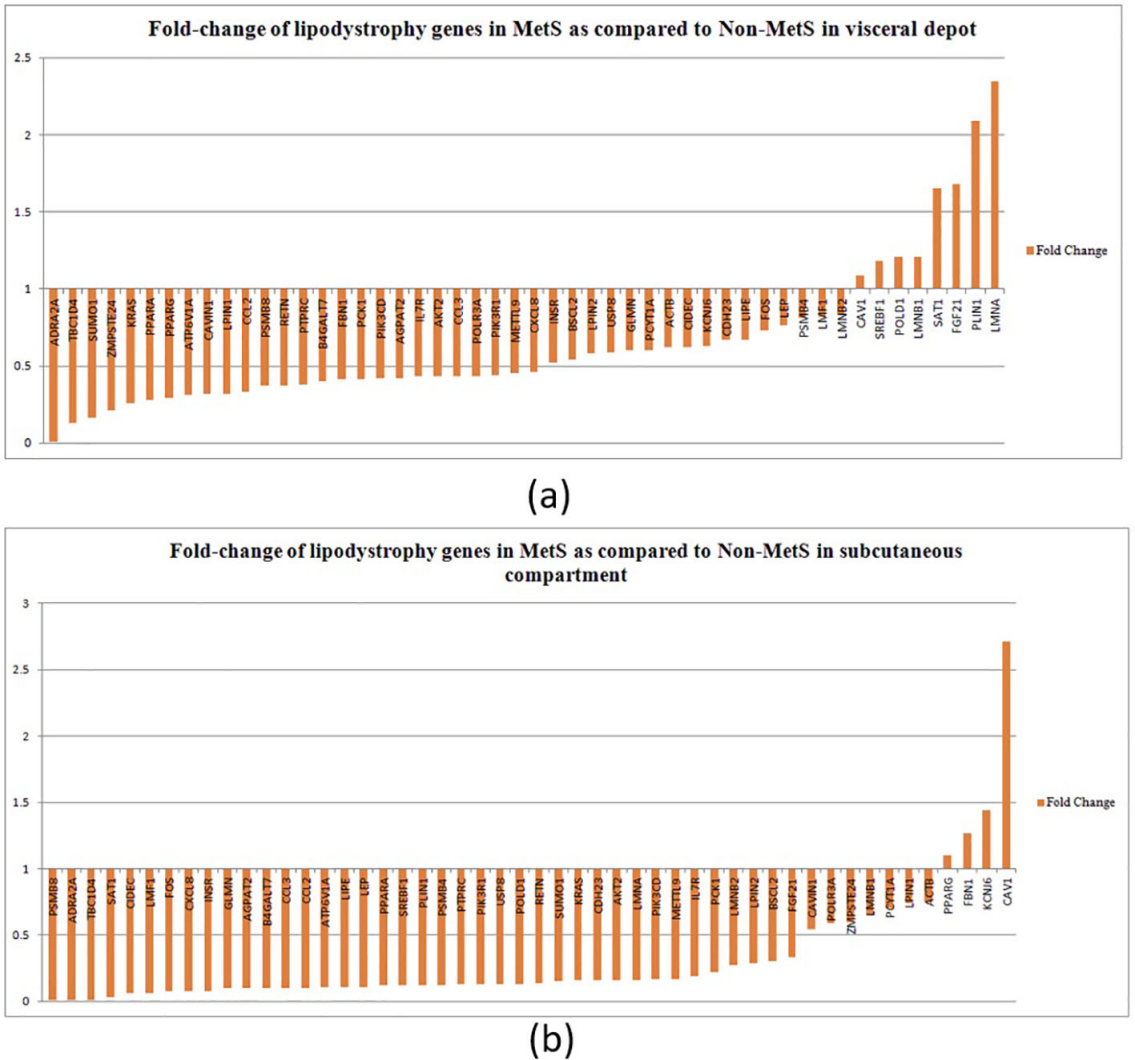
Fold change of lipodystrophy genes in MetS as compared to non-MetS (normalized to one) among individuals in **(A)** the visceral depot and in **(B)** the subcutaneous compartment.

Majority of lipodystrophy genes showed significant downregulation in MetS individuals compared to non-MetS in visceral fat (**Figure 2A**). Interestingly, the upregulated genes *CAV1, POLD1, SAT1, SREBF1, LMNB1, FGF21, LMNA*, and *PLIN1* were those known to disrupt lipid homeostasis, leading to lipid accumulation or abnormal lipid distribution within cells. This could contribute to metabolic disorders such as dyslipidemia, obesity, or lipodystrophy. *CAV1* was found to attenuate the Akt/mTOR pathway and hence alleviate lipid accumulation in non-alcoholic fatty liver disease (53). Overexpression of *SAT1* enhances autophagy, which plays a significant role in lipid metabolism by regulating lipid droplet breakdown and fatty acid oxidation. An earlier study has shown that unbalanced autophagy may lead to type 2 familial partial lipodystrophy (54). Another upregulated gene *PLIN1* encodes perilipin-1, which coats lipid droplets in adipocytes and is involved in droplet formation, triglyceride storage, and lipolysis. One study suggested that *PLIN1* insufficiency causes a favorable metabolic profile (55) and therefore may protect against CVD and conversely its upregulation could lead to MetS.

In the subcutaneous compartment, all except four genes—*PPARG, FBN1, KCNJ6*, and *CAV1*—were found to be downregulated (**Figure 2B**). Upregulation of *PPARγ, KCNJ6*, and *FBN1* promotes lipid storage, improves lipid and glucose metabolism, and attenuates inflammation, making them key regulators of lipid homeostasis. We propose that upregulation of these genes in subcutaneous compartment could be a compensatory mechanism to alleviate disturbed lipid metabolism.

**Figure 3:**
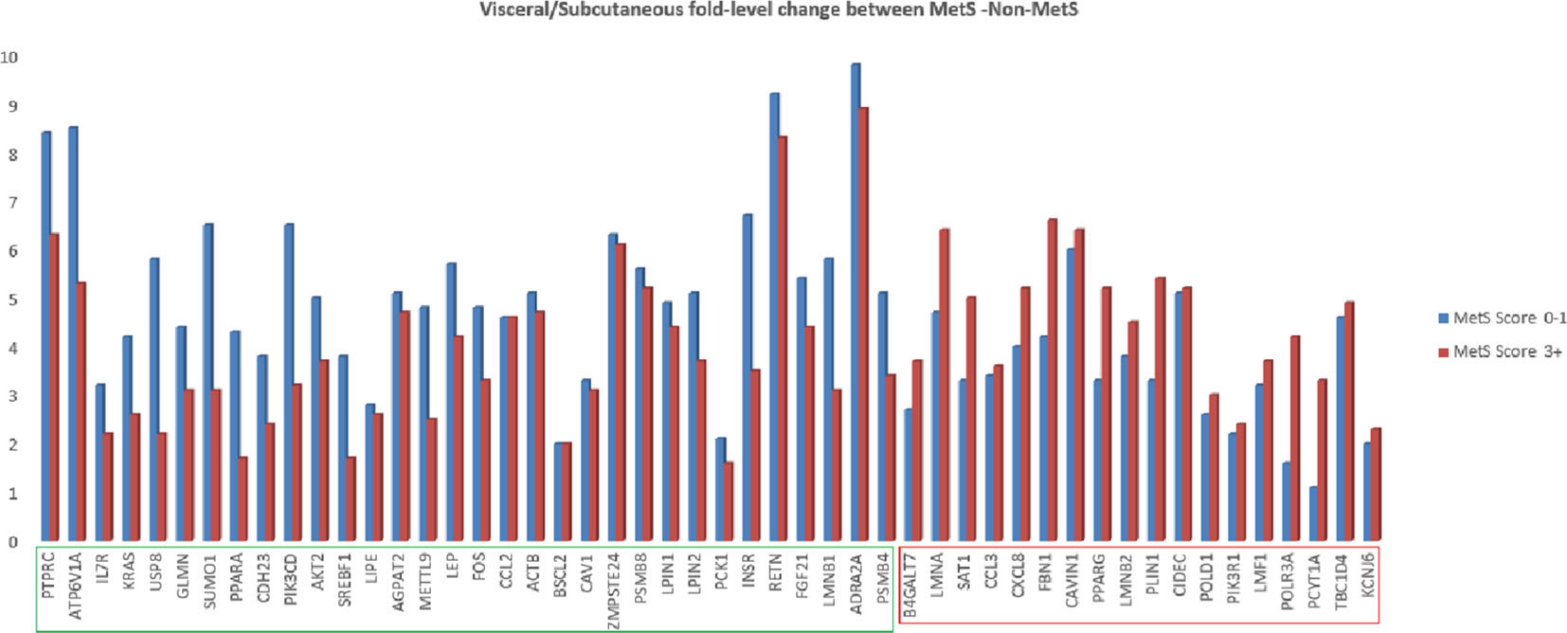
Fold change of lipodystrophy specific genes in abdominal visceral adipose tissue as compared to abdominal subcutaneous adipose tissue in MetS (3+ score) and non-MetS (0–1 score) cohort.

We also analyzed relative fold difference in gene expression between visceral and subcutaneous tissues. All the genes have a relatively higher expression in the visceral depot compared to their subcutaneous counterpart. This relative expression was found to be decreased for 32 genes with increasing MetS score and to be increased for the remaining genes (marked in green and red boxes in **Figure 3**).

Earlier, we have identified modules of co-expressed genes from a gene expression DNA microarray study in abdominal visceral and subcutaneous as well as gluteo-femoral fat depots, which were showing correlation with various clinical and biochemical intermediate traits (48).

On further inspection of these modules for the enrichment of these qPCR gene panels, we could find four genes—*CCL3, CXCL8, FOS*, and *KRAS*—in the gray module, 16 genes—*AGPAT2, AKT2, ATP6V1A, CCL2, CDH23, FBN1, INSR, KCNJ6, LIPE, LMF1, LPIN2, PCK1, PIK3CD, PIK3R1, PPARA*, and *RETN*—in the turquoise module, 13 genes—*ACTB, CAV1, CIDEC, LPIN1, PLIN1, PPARG, PSMB4, PSMB8, SAT1, SUMO1, TBC1D4, USP8*, and *ZMPSTE24—*in the blue module, 2 genes—*LMNA* and *PTPRC*—in the brown module, and 2 genes—*ADRA2A* and *LEP*—in the green module. The association of these 37 genes with diabetes-related intermediate traits reflects the shared molecular etiology of MetS and T2D with lipodystrophy.

### Correlation patterns among gene expressions

Majority of genes (~43–50) have shown a negative correlation with MetS, T2D, and HOMA-IR for both depots, which was in line with differential expression analysis.

In subcutaneous fat, 30 genes compared to 10 genes in the visceral fat showed a positive correlation with HOMA-B, pointing toward a possible relation of insulin secretion with these lipodystrophy genes.

With respect to visceral fat, 40 genes showed a negative correlation, whereas 42 genes showed a positive correlation in subcutaneous with adipocyte size; of them, 28 genes were statistically significant (*p* ~ 0). Earlier, we observed an increasing trend of adipocyte hyperplasia from non-MetS to MetS across subcutaneous and visceral fat, which was statistically significant (*p* < 0.009). **Tables 4**, **5** show the statistically significant correlations (*p* < 0.05) of genes with measured intermediate and biochemical traits in visceral fat and subcutaneous fat, respectively.

**Table 4:** Genes showing statistically significant correlation with measured biochemical and intermediate traits in abdominal visceral depot.

S. no.	Trait	Gene	Correlation	*p*-value
1	Adipocyte size	*TBC1D4*	−0.3	0.0
		*CCL2*	−0.3	0.0
		*ATP6V1A*	−0.2	0.0
		*PIK3R1*	−0.2	0.0
2	C peptide	*CXCL8*	−0.3	0.0
		*PIK3CD*	−0.3	0.0
		*CCL2*	−0.2	0.0
3	Hb1Ac	*ACTB*	−0.3	0.0
		*LMNA*	0.3	0.0
4	HDL	*PLIN1*	0.2	0.0
		*CAVIN1*	0.2	0.0
5	HOMAB	*SAT1*	1.0	0.0
6	HOMAIR	*LMNA*	0.3	0.0
		*LMNB1*	0.3	0.0
		*ACTB*	−0.3	0.0
7	hsCRP	*PPARG*	−0.2	0.0
8	IL.6	*CAV1*	0.4	0.0
		*ACTB*	0.2	0.0
9	LDL	*PIK3CD*	−0.3	0.0
		*PTPRC*	−0.2	0.0
		*LMNA*	0.2	0.0
10	Leptin	*SUMO1*	0.3	0.0
		*PCK1*	0.3	0.0
		*TBC1D4*	0.2	0.1
11	Liver fat	*BSCL2*	0.3	0.0
		*FBN1*	0.3	0.0
12	MetS	*SUMO1*	−0.3	0.0
		*ATP6V1A*	−0.3	0.0
		*CCL2*	−0.3	0.0
		*CCL3*	−0.3	0.0
		*PPARA*	−0.2	0.0
		*CXCL8*	−0.2	0.0
		*PTPRC*	−0.2	0.0
		*LMNA*	0.2	0.0
		*B4GALT7*	−0.2	0.0
		*TBC1D4*	−0.2	0.0
13	TNF-α	*LMNB1*	0.4	0.0
		*LMNA*	0.3	0.0
		*ADRA2A*	−0.3	0.0
14	Total cholesterol	*SUMO1*	−0.3	0.0
		*PIK3CD*	−0.3	0.0
		*CCL2*	−0.3	0.0
		*CCL3*	−0.3	0.0
		*IL7R*	−0.2	0.0
		*PTPRC*	−0.2	0.0
		*TBC1D4*	−0.2	0.0
15	Triglycerides	*FOS*	−0.3	0.0
		*CCL3*	−0.3	0.0
		*PPARA*	−0.3	0.0
		*AKT2*	−0.3	0.0
		*CCL2*	−0.3	0.0
		*METTL9*	−0.3	0.0
		*IL7R*	−0.3	0.0
		*GLMN*	−0.2	0.0
		*B4GALT7*	−0.2	0.0
		*SUMO1*	−0.2	0.0
		*KCNJ6*	−0.2	0.0
16	Visceral fat	*ACTB*	−0.3	0.0
		*LIPE*	0.3	0.0

**Table 5:** Genes showing statistically significant correlation with measured biochemical and intermediate traits in abdominal and peripheral subcutaneous compartments.

S. no.	Trait	Gene	Correlation	*p*
1	Adipocyte size	*SAT1*	−0.4	0.0
		*KCNJ6*	−0.3	0.0
		*PSMB8*	−0.2	0.0
		*TBC1D4*	0.2	0.0
		*CDH23*	−0.2	0.0
		*ACTB*	0.2	0.0
		*PCK1*	0.2	0.0
		*PIK3CD*	0.2	0.0
		*PLIN1*	0.2	0.0
		*CAVIN1*	0.2	0.0
		*IL7R*	0.2	0.0
		*LMNB1*	0.2	0.0
		*SREBF1*	0.2	0.0
		*PTPRC*	0.2	0.0
		*LEP*	0.2	0.0
		*CCL2*	0.2	0.0
		*CAV1*	0.2	0.0
		*INSR*	0.2	0.0
		*GLMN*	0.2	0.0
		*LPIN1*	0.2	0.0
		*PPARA*	0.2	0.0
		*LPIN2*	0.2	0.0
		*AGPAT2*	0.2	0.0
		*FBN1*	0.2	0.0
		*B4GALT7*	0.2	0.0
		*CIDEC*	−0.2	0.0
		*ATP6V1A*	0.2	0.0
		*LMNA*	0.2	0.0
		*KRAS*	0.2	0.0
		*METTL9*	0.2	0.0
		*POLR3A*	0.2	0.0
		*ZMPSTE24*	0.2	0.0
		*PPARG*	0.2	0.0
2	Adiponectin	*SAT1*	0.2	0.0
3	HDL	*TBC1D4*	0.2	0.0
4	TNF-α	*PPARA*	0.6	0.0
		*GLMN*	0.6	0.0
		*POLD1*	0.5	0.0
		*IL7R*	0.4	0.0
		*PIK3CD*	0.4	0.0
		*B4GALT7*	0.4	0.0
		*CCL2*	0.4	0.0
		*AGPAT2*	0.4	0.0
		*USP8*	−0.3	0.0
		*SREBF1*	0.3	0.0
		*CDH23*	−0.3	0.0
		*LEP*	0.3	0.0
		*KCNJ6*	−0.3	0.0
5	Total cholesterol	*PCYT1A*	0.2	0.0
		*PSMB8*	0.3	0.0
		*ADRA2A*	0.2	0.0
6	Triglycerides	*PSMB8*	0.2	0.0
		*LMF1*	0.2	0.0

### MetS patient subgroups based on lipodystrophy-related gene expression profiles

**Figure 4:**
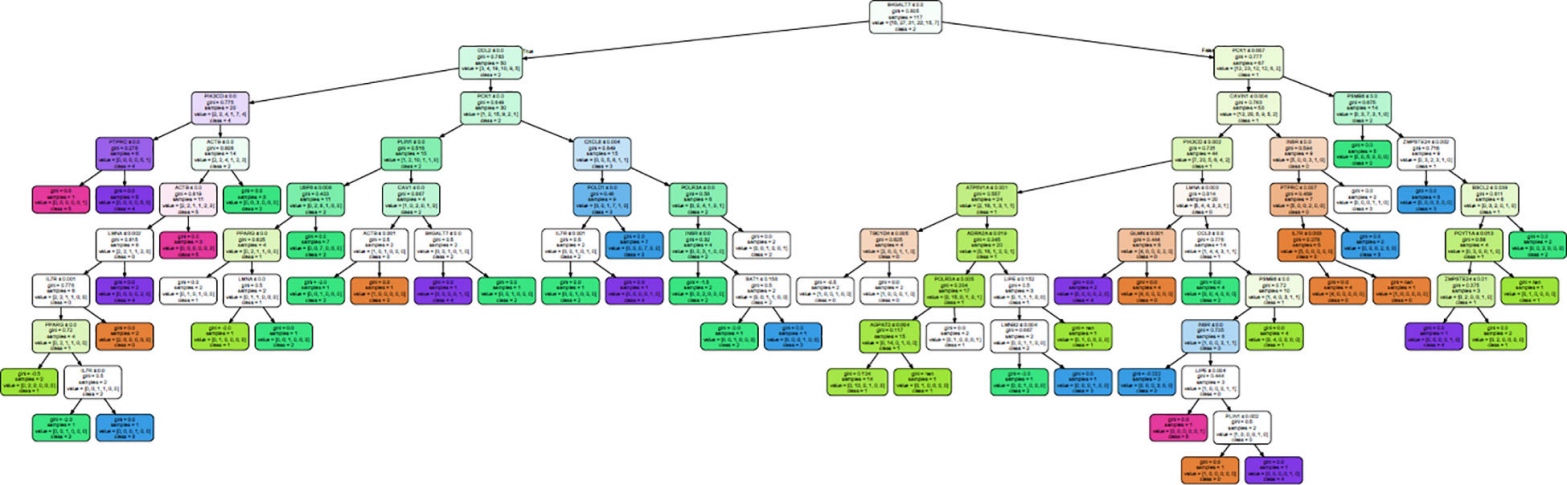
Decision tree generated to classify all the 117 samples into their MetS group in subcutaneous fat.

A decision tree classifier was built for all the 50 genes for subcutaneous fat, and it could segregate all the 117 samples into 50 subgroups, belonging to MetS group 0–5 (**Figure 4**; **Supplementary Figure 1**). Our decision tree could segregate one to two patients along a particular path from root node to MetS class level. This observation, ~2 patients/path (117 patients/50 paths), might be a clinically effective strategy to individualize patients and to impart personalized care.

From this analysis, we were able to conclude that expression of just 28 genes—BSCL2, CAV1, CAVIN1, LMNA, LMNB2, PCYT1A, PLIN1, PPARG, PSMB8, ZMPSTE24, ACTB, AGPAT2, ATP6V1A, B4GALT7, CCL2, CCL3, CXCL8, GLMN, IL7R, INSR, LIPE, PCK1, PIK3CD, POLR3A, PTPRC, SAT1, TBC1D4, and USP8—out of the 50 genes (**Table 6**) is sufficient to classify all the patients. Interestingly, the first 10 genes were also present in the lipodystrophy panel.

**Table 6:** Gene paths identified from the decision tree along with the number of samples in the leaf nodes.

MetS group	No. of samples	Genes	No. of unique genes
0	16	***B4GALT7, PCK1, CAVIN1, INSR, PTPRC, IL7R***	16
		*B4GALT7, PCK1, CAVIN1, INSR, PTPRC, IL7R*	
		*B4GALT7, PCK1, CAVIN1*, ***PIK3CD, LMNA, GLMN***	
		*B4GALT7, PCK1, CAVIN1, PIK3CD*, ***ATP6V1A, TBC1D4***	
		*B4GALT7, PCK1, CAVIN1, PIK3CD, ATP6V1A, TBC1D4*	
		*B4GALT7*, ***CCL2*** *, PCK1*, ***PLIN1, CAV1, ACTB***	
		*B4GALT7, CCL2, PIK3CD*, ***ACTB*** *, ACTB, LMNA, IL7R*	
1	29	***B4GALT7, PCK1, PSMB8, ZMPSTE24, BSCL2, PCYT1A, ZMPSTE24***	25
		*B4GALT7, PCK1*, ***CAVIN1***, ***PIK3CD, LMNA, CCL3, PSMB8***	
		*B4GALT7, PCK1, CAVIN1*, ***PIK3CD*, *ATP6V1A, ADRA2A, LIPE***	
		*B4GALT7, PCK1, CAVIN1, PIK3CD, ATP6V1A, ADRA2A*, ***POLR3A***	
		*B4GALT7, PCK1, CAVIN1, PIK3CD, ATP6V1A, ADRA2A, POLR3A*, ***AGPAT2***	
		*B4GALT7, PCK1, CAVIN1, PIK3CD, ATP6V1A, ADRA2A, POLR3A, AGPAT2*	
		*B4GALT7*, ***CCL2*** *, PCK1*, ***PLIN1, USP8, PPARG, LMNA***	
		*B4GALT7, CCL2, PCK1, PLIN1, USP8, PPARG*	
		*B4GALT7, CCL2, PIK3CD*, ***ACTB*** *, ACTB, LMNA*, ***IL7R*** *, PPARG*	
2	30	*B4GALT7, PCK1, PSMB8, ZMPSTE24, BSCL2*	25
		***B4GALT7, PCK1, PSMB8, ZMPSTE24, BSCL2, PCYT1A***	
		*B4GALT7, PCK1, PSMB8*	
		*B4GALT7, PCK1*, ***CAVIN1, PIK3CD, ATP6V1A, ADRA2A, LIPE, LMNB2***	
		*B4GALT7, CCL2, PCK1*, ***CXCL8, POLR3A***	
		*B4GALT7, CCL2, PCK1, CXCL8, POLR3A*, ***INSR, SAT1***	
		*B4GALT7, CCL2, PCK1, CXCL8, POLR3A, INSR*	
		*B4GALT7, CCL2, PCK1, CXCL8*, ***POLD1***, ***IL7R***	
		*B4GALT7, CCL2, PCK1*, ***PLIN1, CAV1*** *, B4GALT7*	
		*B4GALT7, CCL2, PCK1, PLIN1, CAV1*, ***ACTB***	
		*B4GALT7, CCL2, PCK1, PLIN1*, ***USP8***	
		*B4GALT7, CCL2, PCK1, PLIN1, USP8*, ***PPARG, LMNA***	
		*B4GALT7, CCL2*, ***PIK3CD*** *, ACTB*	
		*B4GALT7, CCL2, PIK3CD, ACTB, ACTB, LMNA, PPARG, IL7R*	
3	24	***B4GALT7, PCK1, PSMB8, ZMPSTE24***	22
		*B4GALT7, PCK1*, ***CAVIN1, INSR***	
		*B4GALT7, PCK1, CAVIN1, INSR*, ***PTPRC***	
		*B4GALT7, PCK1, CAVIN1*, ***PIK3CD, LMNA, CCL3, PSMB8*** *, INSR*	
		*B4GALT7, PCK1, CAVIN1, PIK3CD, LMNA, CCL3, PSMB8, INSR*	
		*B4GALT7, PCK1, CAVIN1, PIK3CD, LMNA, CCL3, PSMB8, INSR*	
		*B4GALT7, PCK1, CAVIN1, PIK3CD, LMNA, CCL3, PSMB8, INSR*	
		*B4GALT7, PCK1, CAVIN1, PIK3CD*, ***ATP6V1A, ADRA2A, LIPE, LMNB2***	
		*B4GALT7, CCL2, PCK1*, ***CXCL8, POLR3A*** *, INSR*, ***SAT1***	
		*B4GALT7, CCL2, PCK1, CXCL8*, ***POLD1***	
		*B4GALT7, CCL2, PIK3CD*, ***ACTB*** *, ACTB, LMNA*, ***PPARG, IL7R***	
4	14	***B4GALT7, PCK1, PSMB8, ZMPSTE24, BSCL2, PCYT1A, ZMPSTE24***	20
		*B4GALT7, PCK1*, ***CAVIN1, PIK3CD, LMNA, CCL3***	
		*B4GALT7, PCK1, CAVIN1, PIK3CD, LMNA*, ***GLMN***	
		*B4GALT7*, ***CCL2*** *, PCK1*, ***CXCL8, POLD1, IL7R***	
		*B4GALT7, CCL2, PCK1*, ***PLIN1, CAV1*** *, B4GALT7*	
		*B4GALT7, CCL2, PIK3CD*, ***ACTB*** *, ACTB, LMNA*	
		*B4GALT7, CCL2, PIK3CD*, ***PTPRC***	
5	4	***B4GALT7, CCL2, PIK3CD, ACTB*** *, ACTB*	5
		*B4GALT7, CCL2, PIK3CD*, ***PTPRC***	
**Total**	**117**		

Unique genes within a MetS group are shown in bold.

## Discussion

In South Asians, the complex genetic trait of T2D is associated with altered expression of several genes that enrich pathways in pathological adipose tissue, with their modules of co-expressed genes showing an association with disease-related intermediate phenotypic traits (42–49). Interestingly, mutations in several of these genes have also been reported to be implicated in the causation of lipodystrophy, a monogenetic disorder of lipid metabolism that also shares phenotypic similarity with T2D and MetS (56, 57). Lipodystrophy and T2D have traditionally been considered two distinct diseases, highlighting the critical role of adipose tissue in metabolic dysfunction. In lipodystrophy, severe MetS develops due to an absolute lack of adipose tissue, whereas in T2D, the metabolic dysfunction arises primarily because adipose tissue function becomes overwhelmed by the stress of overnutrition. Early diagnosis of adipose tissue dysfunction, particularly before the onset of MetS, is therefore crucial in clinical settings to improve outcomes (58). To confirm these observations, we conducted this study by performing large-scale qPCR expression profiling of selected lipodystrophy genes in adipose tissue. The findings of this study can be summarized as follows: (1) the majority of lipodystrophy genes show altered expression in both visceral and subcutaneous compartments in a direction that could adversely influence adipose tissue development or function; (2) the altered expression of these genes also shows an association with T2D, MetS, and IR, and their expression level tends to increase with increasing MetS score; (3) there is a significant overlap between their protein–protein interaction network with T2D interactome; and (4) individualization for prediction of MetS severity based on expression of these lipodystrophy genes is also feasible. Therefore, these findings support the hypothesis that MetS in South Asians share a common pathophysiological and molecular mechanism with congenital lipodystrophy syndromes as its susceptibility genes were found to be differently expressed in T2D patients having MetS. Therefore, functionally, they are more likely to be linked to adipocyte functions in these patients and they might not be specific to congenital lipodystrophy.

Several previous studies describing the striking phenotypic overlap between lipodystrophy and MetS (albeit with different degrees of severity) have suggested that subtle forms of lipodystrophy may be relevant to the pathogenesis of MetS and T2D (36, 36, 59, 60). However, this is a widely hypothetical premise. We previously demonstrated that T2D is associated with altered expression of several lipodystrophy genes in the adipose tissues of South Asians (45). Additionally, we have independently demonstrated a significant overlap between the protein–protein interaction network of lipodystrophy genes and the adipose tissue transcriptome of individuals with T2D (46). The findings of the present study, which revealed the altered expression of lipodystrophy genes in adipose tissue and their association with several MetS, T2D, and IR-related intermediate phenotypic traits, not only confirm our earlier findings but also further point to the role of altered adipose tissue expression of lipodystrophy genes in their pathogenesis. In other words, these findings support the hypothesis that functional defects caused by altered lipodystrophy gene expression in adipose tissue is involved in T2D, MetS, and IR pathogenesis. Another line of evidence to support this premise of a shared molecular thread between congenital lipodystrophy syndrome and T2D and MetS disease is the finding in the present study that various lipodystrophy genes are also part of the pathogenic modules of co-expressed genes identified in previous transcriptomic studies. In other words, the modules of co-expressed genes associated with diabetes and its intermediate phenotypic traits, the so-called “adipose tissue molecular units of dysmetabolism”, also include lipodystrophy genes. Arguably, these findings can only be proven in longitudinal follow-up studies.

Although South Asians as a race exhibit the obesity paradox, its cellular and molecular pathophysiology remains largely unexplored. As discussed previously, we observed that several modules of co-expressed genes in adipose tissue showing association with diabetes and its intermediate phenotypic traits converge on the molecular pathways of adipogenesis and inflammation (47–49, 52). There are also sporadic references supporting the fact that germline mutations in several adipogenesis genes have been identified by linkage analysis in several pedigrees with congenital lipodystrophies and are considered to be the primary drivers of the pathophysiology of these syndromes. Therefore, the two instances of the obesity paradox, i.e., congenital lipodystrophies and Asian Indian MetS and T2D, appear to converge in the cellular process of adipogenesis.

This shared functional molecular pathway between T2D, IR, MetS, and lipodystrophy has several implications. Firstly, the mechanisms and pathways of severe metabolic defects underlying congenital lipodystrophies are well deciphered, and their altered expression and representation in the modules of co-expressed genes in adipose tissue in patients with diabetes suggests their role in the pathogenesis of T2D and adipocentric metabolic dysfunction underlying MetS (i.e., MetS disease or adiposopathy) on the same lines. The significance of this observation is that altered expression of lipodystrophy genes in various adipose compartments can serve as a template for studying the precise molecular mechanisms of IR, T2D, and MetS disease. Investigation in both directions, i.e., the mechanisms of their regulation and the cellular and metabolic pathways regulated by them, can decipher the genome-to-phenome pathways and mechanisms of T2D and MetS disease. This view is also important considering our finding that altered expression of only a few genes is a major determinant of clinical MetS. Therefore, these findings highlight the oligogenic nature of complex traits of T2D and MetS disease (61, 62). Secondly, the constituent genes in the pathological “modules of co-expressed genes”, which are otherwise not known to be implicated in the pathogenesis of congenital lipodystrophy, could be explored for mutations in pedigrees with unexplained congenital lipodystrophies. Thirdly, as the genome-to-phenome pathways of congenital lipodystrophies are well understood, the primary role of adipose tissue dysfunction in the pathogenesis of the severe underlying metabolic dysfunction is well established. Therefore, on the same lines, altered expression of these genes in adipose tissue and their association with IR, T2D, and MetS support the concept of “MetS disease” or the so-called “adiposopathy” as a primary disease and the components of “clinical MetS” as its clinical manifestations (63–66). However, this concept needs to be validated in longitudinal and intervention studies in the future.

We further attempted to identify hallmark genes that could be decisive in stratifying “clinical MetS” individuals and constructed a decision tree using machine learning. This decision tree requires only 20 genes (among the 50 genes) to classify “clinical MetS”. Nine of them were also included in the monogenic lipodystrophy syndrome diagnostic gene panel; this further points out how ancient lipid dysfunction with vivid molecular etiology could be shaped by various evolutionary processes and transformed into present-day IR, T2D, and MetS disease. This picture—however obscure—also promises to deliver fundamental drug targets that enable personalized T2D and MetS therapy in the future.

There are several limitations to this study. This study was conducted at a single center with a relatively small sample size. However, owing to ethical considerations, it was difficult to obtain a large sample size. Second, the study was cross-sectional in design; thus, causal inferences could not be drawn. Longitudinal studies performed at different stages of natural T2D and MetS disease can only help confirm these findings, but such studies are challenging to perform. Finally, it is important to determine whether gene expression can be reversed by a healthy lifestyle, including the intake of low-calorie diets and/or increased physical activity.

## Data Availability

The original contributions presented in the study are included in the article/**Supplementary Material**. Further inquiries can be directed to the corresponding author.
