# Supplementary material for: Exploring lipodystrophy gene expression in adipocytes: unveiling insights into the pathogenesis of insulin resistance, type 2 diabetes, and clustering diseases (metabolic syndrome) in Asian Indians
Source: Front Endocrinol (Lausanne). 2024 Oct 9;15:1468824. doi: 10.3389/fendo.2024.1468824 (PMC11496143; doi:10.3389/fendo.2024.1468824)
Supplement: Supplementary file 1 [file DataSheet1.zip › S1.pdf]

## Table 1. Evidences for the role of genes in lipodystrophy/adipose biology extrcated from PubMed

| *S. No.* | *Gene Symbol* | *Gene Name* | *Association Type* | *Sentence* | *PMID* |
|---|---|---|---|---|---|
| 1 | *ADRA2A* | Adrenoceptor alpha 2A | Biomarker | We conclude that heterozygous p.Leu68Phe ADRA2A mutation causes a rare atypical FPLD, most likely by inducing excessive lipolysis in some adipose tissue depots. | 27376152 |
| 2 | *AGPAT2* | 1-Acylglycerol-3-phosphate O-acyltransferase 2 | Biomarker | We conclude that heterozygous p.Leu68Phe ADRA2A mutation causes a rare atypical FPLD, most likely by inducing excessive lipolysis in some adipose tissue depots. | 27376152 |
| 3 | *AKT2* | AKT serine/threonine kinase 2 | Biomarker | Clinical characteristics and efficacy of pioglitazone in a Japanese diabetic patient with an unusual type of familial partial lipodystrophy. | 19793595 |
| 4 | *BSCL2* | BSCL2, seipin lipid droplet biogenesis associated | GeneticVariation | Our findings demonstrate that disruption of Bscl2 specifically in developing adipocytes is sufficient to cause the early-onset generalised lipodystrophy observed in patients with mutations in BSCL2. | 29459250 |
| 5 | *CAV1* | Caveolin 1 | Biomarker | Clinical characteristics and efficacy of pioglitazone in a Japanese diabetic patient with an unusual type of familial partial lipodystrophy. | 19793595 |
| 6 | *CAVIN1* | Caveolae associated protein 1 | Biomarker | Mutations in AGPAT2, BSCL2, CAV1, and PTRF have been reported in congenital generalized lipodystrophy and in LMNA, PPARG, AKT2, and PLIN1 in FPL. | 21865368 |
| 7 | *CCL2* | C-C motif chemokine ligand 2 | AlteredExpression | Inflammatory gene expression was increased in HAART-lipodystrophy: CD68 4.5-fold (P = 0.000013), tumor necrosis factor (TNF)-alpha 2-fold (P = 0.0094), chemokine (C-C motif) ligand (CCL) 2 2.5-fold (P = 0.0024), CCL3 7-fold (P = 0.0000017), integrin alphaM (ITGAM) 3-fold (P = 0.00067), epidermal growth factor-like module containing, mucin-like, hormone receptor-like (EMR)1 2.5-fold (P = 0.0038), and a disintegrin and metalloproteinase domain (ADAM)8 3.5-fold (P = 0.00057) higher in the HAART+LD+ compared with the HAART+LD- group. mRNA concentration of CD68 (r = 0.37, P = 0.019), ITGAM (r = 0.35, P = 0.025), CCL2 (r = 0.39, P = 0.012), and CCL3 (r = 0.54, P = 0.0003) correlated with liver fat content. | 18430964 |
| 8 | *CCL3* | C-C motif chemokine ligand 3 | AlteredExpression | | 18430964 |
| 9 | *PPARA* | Peroxisome proliferator activated receptor alpha | AlteredExpression | | 18430964 |
| 10 | *CIDEC* | Cell death inducing DFFA like effector c | GeneticVariation | Partial lipodystrophy and insulin resistant diabetes in a patient with a homozygous nonsense mutation in CIDEC. | 20049731 |
| 11 | *CXCL8* | C-X-C motif chemokine ligand 8 | AlteredExpression | No differences were found relating IL-1α, IL-1β, IL-2, IL-6 and IL-8 levels in lipodystrophy vs. non-lipodystrophy. | 28189545 |
| 12 | *FBN1* | Fibrillin 1 | GeneticVariation | Further evidence for a marfanoid syndrome with neonatal progeroid features and severe generalized lipodystrophy due to frameshift mutations near the 3' end of the FBN1 gene. | 21594992 |
| 13 | *FGF21* | Fibroblast growth factor 21 | AlteredExpression | We hypothesized that muscle FGF-21 mRNA would be altered in HIV patients with lipodystrophy. | 23533568 |
| 14 | *FOS* | Fos proto-oncogene, AP-1 transcription factor subunit | GeneticVariation | A mutation in the c-fos gene associated with congenital generalized lipodystrophy. | 23919306 |
| 15 | *GLMN* | Glomulin, FKBP associated protein | Biomarker | Role of FAP48 in HIV-associated lipodystrophy. | 22678819 |
| 16 | *IL7R* | Interleukin 7 receptor | Biomarker | This was also the case for IL-7R expressing $CD8^+$ T cells ($CD127^+$) for lipoatrophy β = 12.8, P = 0.003, and for central fat accumulation β = 9.45, P = 0.016. | 24129368 |
| 17 | *INSR* | Insulin receptor | GeneticVariation | Both patients had partial lipodystrophy and a novel heterozygous missense mutation (Asn<sup>1137</sup> → Lys<sup>1137</sup>) in the insulin receptor gene. | 31102683 |
| 18 | *KCNJ6* | Potassium voltage-gated channel subfamily J member 6 | GeneticVariation | Here, we describe a fourth case of a human with a de novo KCNJ6 (GIRK2) mutation, who presented with clinical findings of severe hyperkinetic movement disorder and developmental delay, similar to the Keppen-Lubinsky syndrome but without lipodystrophy. | 29852244 |
| 19 | *LEP* | Leptin | AlteredExpression | Low leptin levels in CGL-1 support the claim that subjects are hypermetabolic and hyperphagic.The present study examines this claim. | 18640396 |
| 20 | *LMF1* | Lipase maturation factor 1 | Biomarker | Eight different genetic loci, including 1-acylglycerol-3-phosphate-O-acyltransferase 2, Berardinelli-Seip congenital lipodystrophy 2, caveolin 1, lamin A/C, peroxisome proliferator-activated receptor gamma, v-AKT murine thymoma oncogene homolog 2, zinc metalloprotease and lipase maturation factor 1 have been described linked to different lipodystrophy syndromes. | 19494770 |

| | | | | | |
|---|---|---|---|---|---|
| 21 | *LMNA* | Lamin A/C | GeneticVariation | Whole exome sequencing revealed de novo heterozygous LMNA p.T10I mutation, indicating generalized lipodystrophy-associated progeroid syndrome, which is a newly identified subtype of atypical progeroid syndrome characterized by severe metabolic abnormalities. | 31708526 |
| 22 | *LMNB1* | Lamin B1 | GeneticVariation | Duplication in LMNB1 encoding lamin B1 causes autosomal dominant leukodystrophy and mutations in LMNB2 encoding lamin B2 are associated with acquired partial lipodystrophy. | 17467691 |
| 23 | *LMNB2* | Lamin B2 | GeneticVariation | Duplication in LMNB1 encoding lamin B1 causes autosomal dominant leukodystrophy and mutations in LMNB2 encoding lamin B2 are associated with acquired partial lipodystrophy. | 17467691 |
| 24 | *LPIN1* | Lipin 1 | AlteredExpression | The aim of this study was to analyze LPIN1 adipose tissue gene expression levels in 3 clinical insulin-resistant conditions-obesity, type 2 diabetes mellitus, and human immunodeficiency virus (HIV)-associated lipodystrophy-and its relationship with adipogenic and inflammatory markers. | 17950103 |
| 25 | *LPIN2* | Lipin 2 | Biomarker | Lipin 2 (LPIN2), a candidate gene for lipodystrophy, maps in proximity to this locus. | 15862761 |
| 26 | *METTL9* | Methyltransferase like 9 | Biomarker | Our results establish the three mammalian lipin proteins as PAP1 enzymes and explain the biochemical basis for lipodystrophy in the lipin-1-deficient fld mouse. | 17158099 |
| 27 | *PCK1* | Phosphoenolpyruvate carboxykinase 1 | GeneticVariation | (4) An adipose-specific knockout of Pck1 results in a fraction of the mice developing lipodystrophy due to lost glyceroneogenesis and a consequent decrease in fatty acid re-esterification. | 17709878 |
| 28 | *PCYT1A* | Phosphate cytidylyltransferase 1, choline, alpha | GeneticVariation | Mutations in the PCYT1A gene have been recently linked to two different phenotypes: one characterized by spondylometaphyseal dysplasia and cone-rod dystrophy (SMD-CRD) and the other by congenital lipodystrophy, severe fatty liver disease, and reduced HDL cholesterol without any retinal or skeletal involvement. | 28272537 |
| 29 | *PIK3CD* | Phosphatidylinositol-4,5-bisphosphate 3-kinase catalytic delta | AlteredExpression | Normal PI3K activity is critical for adipose differentiation and insulin signaling; the mutated PIK3R1 therefore provides a unique link among lipodystrophy, growth, and insulin signaling. | 23810379 |
| 30 | *PIK3R1* | Phosphoinositide-3-kinase regulatory subunit 1 | AlteredExpression | Normal PI3K activity is critical for adipose differentiation and insulin signaling; the mutated PIK3R1 therefore provides a unique link among lipodystrophy, growth, and insulin signaling. | 23810379 |
| 31 | *PLIN1* | Perilipin 1 | Biomarker | Mutations in AGPAT2, BSCL2, CAV1, and PTRF have been reported in congenital generalized lipodystrophy and in LMNA, PPARG, AKT2, and PLIN1 in FPL. | 21865368 |
| 32 | *PPARG* | Peroxisome proliferator activated receptor gamma | GeneticVariation | Biallelic mutations at PPARG cause a congenital, generalized lipodystrophy similar to the Berardinelli-Seip syndrome. | 24980513 |
| 33 | *PTPRC* | Protein tyrosine phosphatase, receptor type C | Biomarker | Expression of adipogenic transcription factors, peroxisome proliferator-activated receptor gamma co-activator 1, IL-6 and CD45 in subcutaneous adipose tissue in lipodystrophy associated with highly active antiretroviral therapy. | 12891061 |
| 34 | *RETN* | Resistin | Biomarker | The aim of this study was to correlate levels of leptin, adiponectin, and resistin between HIV/AIDS patients with LD and without lipodystrophy (non-LD), as well as between subgroups of LD [lipoatrophy (LA), lipohypertrophy (LH), and mixed fat redistribution (MFR)] and non-LD patients. | 28339344 |
| 35 | *SAT1* | Spermidine/spermine N1-acetyltransferase 1 | Biomarker | Thus, the aim of the study was to search for an association between the presence of clinical lipodystrophy (LD), visceral and subcutaneous abdominal adipose tissue amount (VAT and SAT), and peripheral T-cell immune phenotypes. | 24129368 |
| 36 | *SREBF1* | Sterol regulatory element binding transcription factor 1 | Biomarker | A novel interaction between lamin A and SREBP1: implications for partial lipodystrophy and other laminopathies. | 11929849 |

| | | | | | |
|---|---|---|---|---|---|
| 37 | *SUMO1* | Small ubiquitin-like modifier 1 | GeneticVariation | On the basis of the position of K486 on the lamin A Ig-fold, we hypothesize the SUMO1 E2 enzyme recognizes a folded structure-dependent motif that includes residues genetically linked to familial partial lipodystrophy (FPLD). | 23243001 |
| 38 | *ZMPSTE24* | Zinc metallopeptidase STE24 | GeneticVariation | MAD patients characterized by generalized lipodystrophy (type B) affecting the face as well as extremities and severe progressive glomerulopathy present heterozygous compound mutations in the ZMPSTE24 gene. | 25286833 |
