# Supplementary figures and images for: Exploring lipodystrophy gene expression in adipocytes: unveiling insights into the pathogenesis of insulin resistance, type 2 diabetes, and clustering diseases (metabolic syndrome) in Asian Indians
Source: Front Endocrinol (Lausanne). 2024 Oct 9;15:1468824. doi: 10.3389/fendo.2024.1468824 (PMC11496143; doi:10.3389/fendo.2024.1468824)

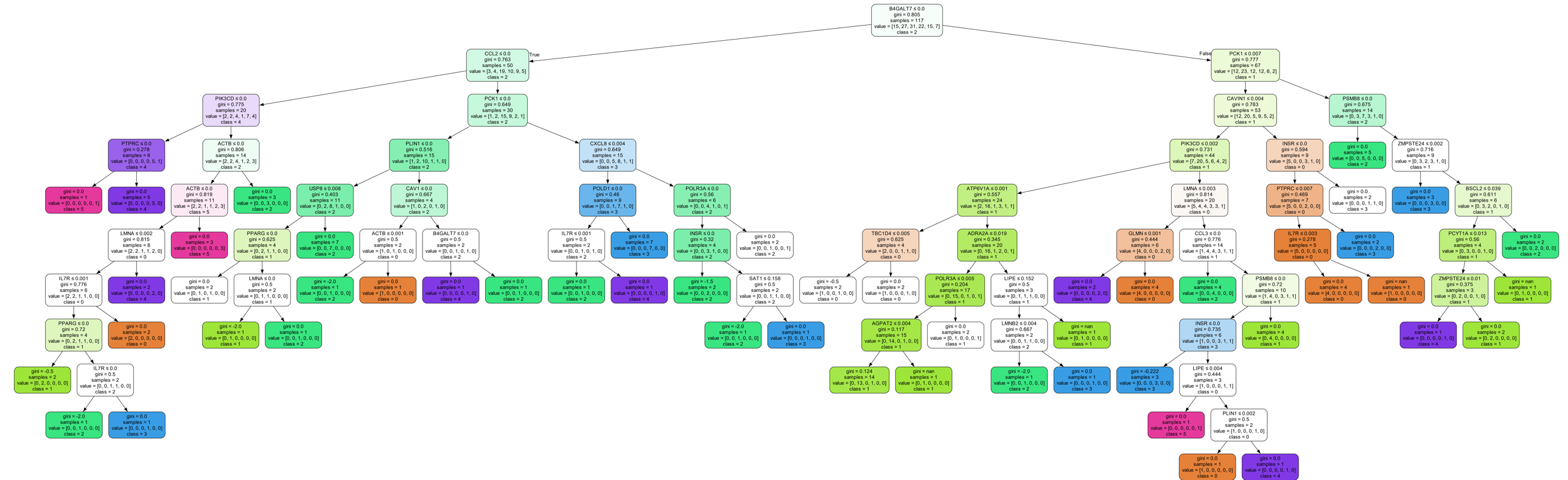

Supplement: Supplementary file 1 [file DataSheet1.zip › S2.pdf.pdf]
